# Characterization and quantification of angiogenesis in rheumatoid arthritis in a mouse model using μCT

**DOI:** 10.1186/1471-2474-15-298

**Published:** 2014-09-06

**Authors:** Svitlana Gayetskyy, Oleg Museyko, Johannes Käßer, Andreas Hess, Georg Schett, Klaus Engelke

**Affiliations:** Institute of Medical Physics, University of Erlangen-Nuremberg, Henkestr. 91, 91052 Erlangen, Germany; Institute of Pharmacology and Toxicology, University of Erlangen-Nuremberg, Erlangen, Germany; Department of Internal Medicine 3 and Institute for Clinical Immunology, University of Erlangen–Nuremberg, Erlangen, Germany

**Keywords:** Angiogenesis, Vascularization, Inflammatory arthritis, μCT, Vessel segmentation

## Abstract

**Background:**

Angiogenesis is an important pathophysiological process of chronic inflammation, especially in inflammatory arthritis. Quantitative measurement of changes in vascularization may improve the diagnosis and monitoring of arthritis. The aim of this work is the development of a 3D imaging and analysis framework for quantification of vascularization in experimental arthritis.

**Methods:**

High-resolution micro-computed tomography (μCT) was used to scan knee joints of arthritic human tumor necrosis factor transgenic (hTNFtg) mice and non-arthritic wild-type controls previously perfused with lead-containing contrast agent Microfil MV-122. Vessel segmentation was performed by combination of intensity-based (local adaptive thresholding) and form-based (multi-scale method) segmentation techniques. Four anatomically defined concentric spherical shells centered in the knee joint were used as analysis volumes of interest. Vessel density, density distribution as well as vessel thickness, surface, spacing and number were measured. Simulated digital vessel tree models were used for validation of the algorithms.

**Results:**

High-resolution μCT allows the quantitative assessment of the vascular tree in the knee joint during arthritis. Segmentation and analysis were highly automated but occasionally required manual corrections of the vessel segmentation close to the bone surfaces. Vascularization was significantly increased in arthritic hTNFtg mice compared to wild type controls. Precision errors for the morphologic parameters were smaller than 3% and 6% for intra- and interoperator analysis, respectively. Accuracy errors for vessel thickness were around 20% for vessels larger than twice the resolution of the scanner.

**Conclusions:**

Arthritis-induced changes of the vascular tree, including detailed and quantitative description of the number of vessel branches, length of vessel segments and the bifurcation angle, can be detected by contrast-enhanced high-resolution μCT.

**Electronic supplementary material:**

The online version of this article (doi:10.1186/1471-2474-15-298) contains supplementary material, which is available to authorized users.

## Background

Rheumatoid arthritis is a chronic inflammatory joint disease characterized by proliferation of the synovial tissue and influx of immune cells into the joint, which results in articular cartilage and bone degradation [1, 2]. The complex changes in tissue architecture during inflammatory arthritis rely on a profound reorganization of the vasculature of the joint, which governs cell in- and efflux as well as the proliferation, an invasion of inflammatory tissue into the cartilage and the bone [3].

Angiogenesis is the formation of new blood vessels by sprouting and pruning of existing vessels [4]. This process occurs physiologically in the embryo and during pathology such as wound healing but also in conjunction with more chronic processes such as cancer or chronic inflammatory disease. During inflammatory arthritis new blood vessels are continuously built in the synovial tissue in order to support the high metabolic activity of the inflammatory tissue. These microvessels allow the migration of immune cells into the inflammatory tissue but also facilitate the invasion of inflammatory synovial tissue into bone and cartilage [5–7]. Furthermore, the proliferation of resident mesenchymal cells as well as the high metabolic activity of immune cells in the context of joint inflammation requires an energy supply, which essentially depends on an appropriate perfusion enabled by microvessels.

Most of the studies to date have assessed the vascularization of the arthritic joints by histology, which has limitations based on its 2-dimesional character. Histologic examination of the blood vessels hardly allows understanding changes of the vascular tree and the vascular architecture during arthritis [8, 9]. We therefore aimed to visualize and quantify the anatomical basis of the vascularization of the joint during arthritis. We employed high-resolution contrast-enhanced μCT imaging in order to get a comprehensive picture of the changes of the vasculature in the inflamed joint.

The problem to quantify angiogenesis and vascularization is relevant beyond arthritis and similar techniques also using high resolution μCT imaging have recently been developed for tumor research [10, 11]. In this methodologically-orientated study we developed dedicated imaging and image analysis protocols for arthritis and applied them in a preclinical model.

## Methods

### μCT imaging

In order to optimize the contrast between blood vessels and surrounding soft tissue, the lead-containing contrast agent Microfil MV-122 (http://www.flowtech-inc.com/microfil.htm) was administered into the aorta of wild type and human tumor necrosis factor transgenic (hTNFtg) mice (each N = 7) prior to imaging [12]. All animal experiments were according to the German Laws for Animal Protection (Tierschutzgesetz) and Animal Experiments (Tierversuchsgesetz) and approved by the responsible institutions (Bundesinstitut für Risikobewertung).

Systemic perfusion was started by anesthetizing the animals with 100 μl Ketamin (100 mg/ml, Pfizer) and 50 μl Xylacin (20 mg/ml, Bayer). After deep anesthesia, checking that no withdrawal reflexes were present, the chest was opened and the left heart chamber punctuated by a 20G cannula. The perfusion was started by flushing the circulation system with 20 ml NaCl 0.9% + 2000 IE heparin with a pump (Ismatec ISM597D). Immediately after begin the circulation system was relieved by cutting large parts of the liver. After perfusion with 20 ml NaCl the perfusant was switched to 20 ml buffered 10% formalin. All solutions were delivered at 37°C and with a pressure of 130 mmHG. Afterwards the manual perfusion was performed with yellow Microfil® (MV-122, 4 ml compound + 5 ml diluent + 0,5 ml curing agent). Finally the aorta and portal vein were ligated and the mice were placed at 4°C for 24 h.

After hardening of the contrast agent, the knee joints were excised. The samples were scanned with a custom built high-resolution μCT scanner developed at the Institute of Medical Physics [13] using 70 kV and 140 μAs. Datasets were reconstructed with an isotropic voxel size of 15 microns using 600 projections. Scan times were approximately one hour per sample.

### Quantification of vascularization

A multi step 3D segmentation procedure described in detail in the Appendix was developed to separate vessels from bone and soft tissue. Two different techniques, both originally developed for the analysis of trabecular bone architecture were used for the quantification of the vascularization in four concentric volumes of interest (VOIs) centered in the knee joint. Vessel density (vessel volume per tissue volume, VV/TV) was computed in analogy to bone volume per tissue volume (BV/TV). Based on the assumption of a rod-like vessel geometry [14] vessel thickness (V.Th_2D_), vessel separation (V.Sp_2D_) and vessel number (V.N_2D_) were calculated as averages from a slice by slice analysis.

The second technique [15], which does not require specific model assumptions, was applied to calculate vessel surface (*V.S*) and the direct vessel thickness (*V.Th*_*3D*_), determined as a distance transform of the centerline voxels of the vessels obtained by a thinning algorithm. Table 1 summarizes the quantitative parameters used in this study. It should be noted that the vessel thickness represents the contrast filled lumen diameter of the vessel but for readability we will continue to use the term vessel thickness.

**Table 1:** **Parameters to quantify vascularization**

Model independent direct 3D parameters	
Vessel density (vessel volume per total volume)	*VV/TV*
Vessel surface	*VS*
Vessel thickness	*V.Th*_*3D*_
Rod-model based parameters	
Vessel thickness	$V.Th_{2D}=4/\left(\frac{\mathrm{VS}}{\mathrm{VV}}\right)$
Vessel spacing	$V.Sp_{2D}=V.Th_{2D}\cdot \left(\sqrt{\frac{4/\pi \cdot \mathrm{TV}}{\mathrm{VV}}}-1\right)$
Vessel number	$V.N_{2D}=\sqrt{\left(\frac{4}{\pi }\cdot \frac{\mathrm{VV}}{\mathrm{TV}}\right)/V.Th_{2D}}$

### Vascular tree models

To estimate the accuracy of computed parameters, CT datasets of 10 different digital vessel tree models were simulated. Each tree consisted of various vessel segments of different orientation and diameter. The vessel tree models were used to compare the different segmentation methods. In addition to the local adaptive threshold (LAT) and multi scale (MS) segmentation techniques described in the Appendix, a simple global threshold (GT) segmentation was also used to classify vessels. In addition, the digital vascular tree models were used to compare the accuracy of the two vessel thickness parameters. The true thickness is known from the model generation.

### Analysis reproducibility

Intra- and inter-operator reanalysis precision calculated as root mean square coefficient of variation *CV*_*rms*_[16] was determined using datasets from 6 different mice. For intraoperator precision one operator analyzed each 6 datasets three times. Inter-operator precision was determined from the analysis of the six datasets by three different operators.

## Results

### Construction of digital vascular models

**Figure 1:**
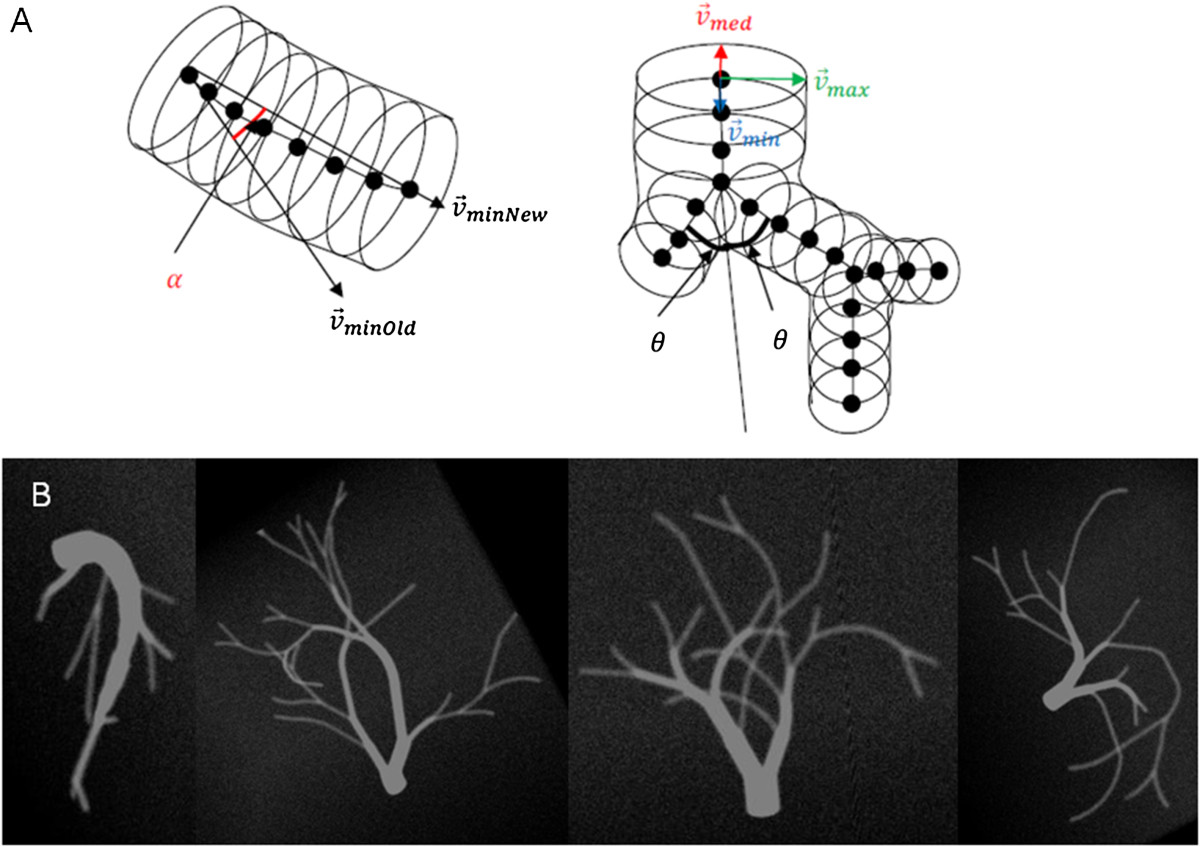
**Generation of digital vessel model. (A)** Geometrical construction, **(B)** examples of different vessel tree models.

The generation of the digital vascular models is illustrated in Figure 1. An individual vessel segment consisted of oriented circles to approximate a curved cylinder. The curving and branching angles α and θ and the vessel diameter were randomly chosen based on certain boundary conditions, for example that branching reduced the vessel diameter but total cross-sectional vessel area before and behind the branch was constant. To simulate realistic data, the generated vascular vessel tree datasets were then smoothed by Gaussian blurring and noise was added.

### Accuracy of vessel volume measurement

**Figure 2:**
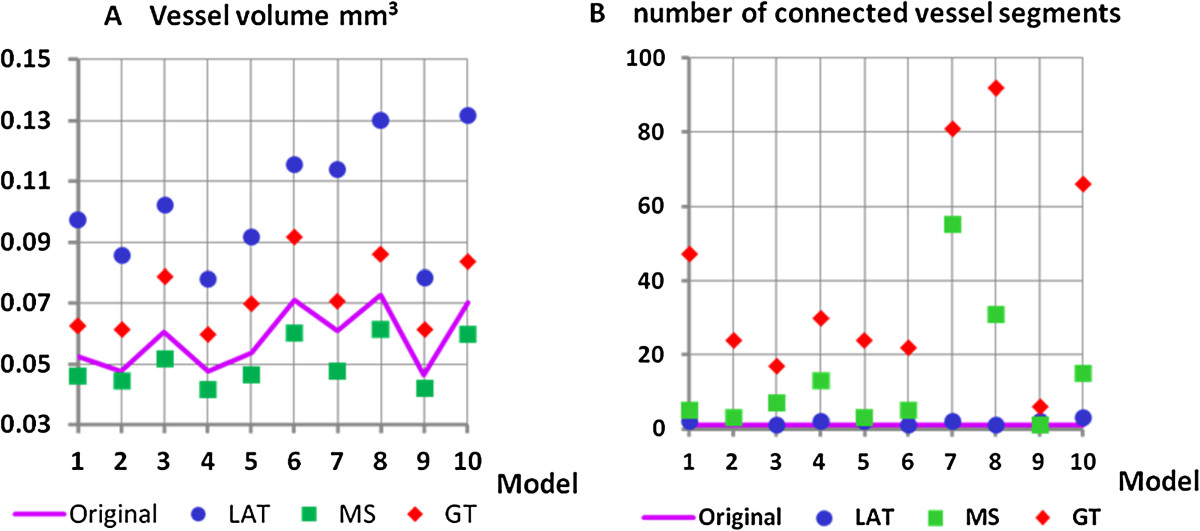
**Accuracy dependence on segmentation. (A)** vessel volume **(B)** vessel number measured as number of connected vessel segments. LAT: local adaptive threshold, MS: multi-scale and GT: global threshold based segmentation.

We first addressed the effect of the three different segmentation techniques (GT, MS, LAT) on vessel volume and number (Figure 2). Most accurate results for vessel volume (Figure 2A) were obtained with the MS segmentation, whereas the LAT method consistently overestimated vessel volume by a factor two. In contrast, the MS segmentation in most models could not reproduce the vessel number of the model. Each model consisted of one connected structure, but with MS on average more than 10 separate entities were measured, i.e. more than 10 disruptions of the vessel tree were falsely obtained. This was not the case with the LAT segmentation, which almost perfectly preserved the connectivity.

### Accuracy of vessel thickness measurement

**Figure 3:**
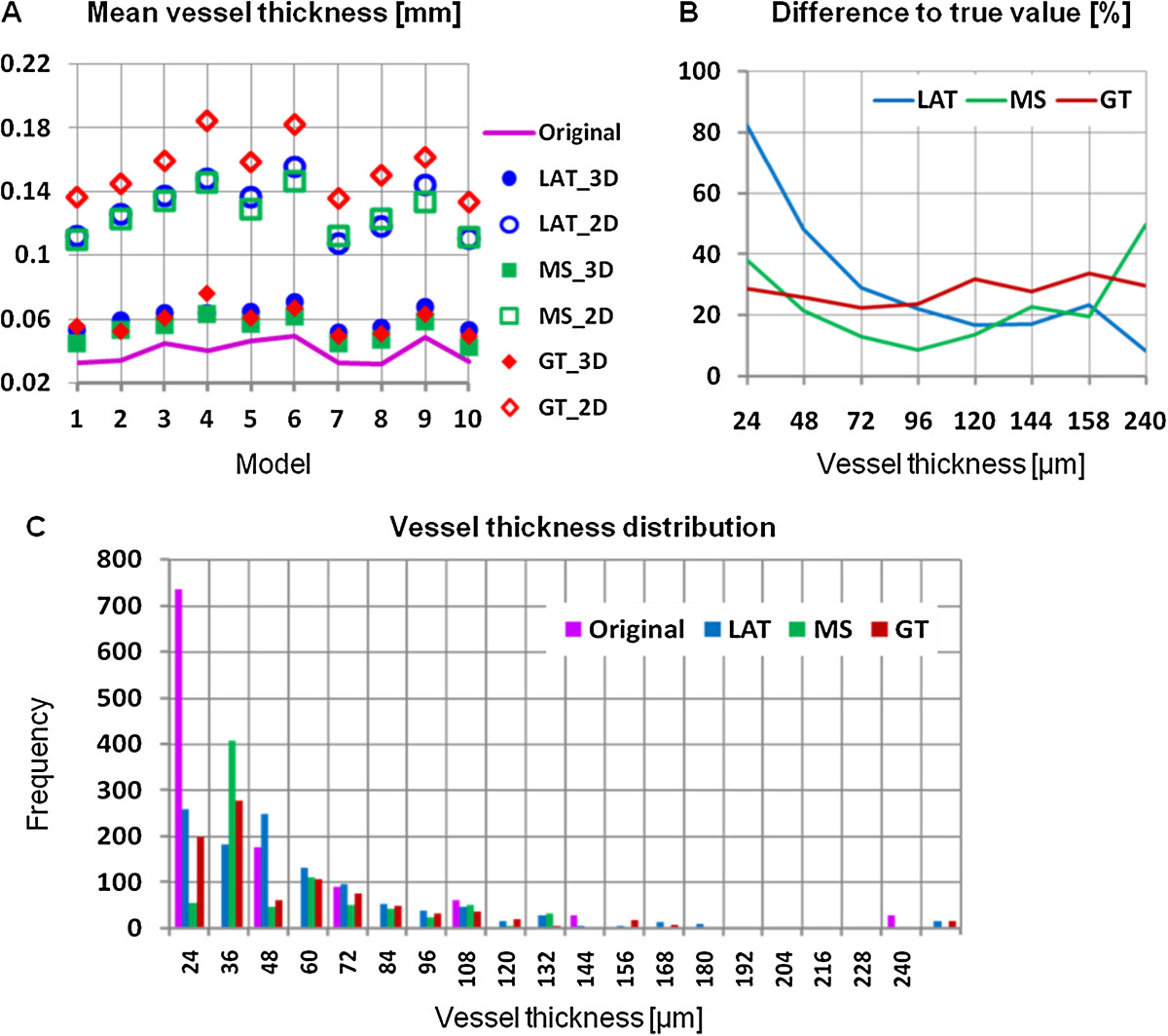
**Accuracy of vessel thickness determined with the digital vascular tree models. (A)** 2D and 3D vessel thickness compared to the true value for different segmentation algorithms. LAT: local adaptive threshold, MS: multi-scale and GT: global threshold based segmentation. **(B)** Difference between measured and true vessel thickness (3D direct measurement) depending on vessel size. Results are averaged over all 10 models. **(C)** Distribution of true vessel thickness values in digital model compared to the results (3D direct measurement) of the three vessel segmentation algorithms. Results are shown for digital model number 1.

Next we addressed the effect of the three segmentation techniques on vessel thickness, either determined by a rod-model measuring *V.Th*_*2D*_ or by direct measure of *V.Th*_*3D*_. Obviously, the accuracy error was much higher in *V.Th*_*2D*_ compared to *V.Th*_*3D*_. Nevertheless, even the direct measurement considerably overestimated the true vessel thickness. The GT segmentation technique resulted in accuracy errors between 20% and 35% independent of vessel thickness. Accuracy errors were smaller (below 20%) except for very thin (<40 μm) or very thick vessels (>150 μm) when using the MS segmentation technique. Finally, the LAT segmentation performed similar to MS in the thickness range between 100 to 150 μm but below a thickness of 50 μm resulted in increasing accuracy errors with decreasing vessel thickness (Figure 3B). This is confirmed in Figure 3C, which shows the V.Th_3D_ distribution of the articular blood vessels. In the digital model a voxel size of 12 μm was used but vessels had a minimum radius of one voxel, thus only vessel diameters of more than 24 μm appear in the digital models. The simulated CT images also had a voxel size of 12 μm but due to partial volume artifacts and noise, in particular cortical thickness is overestimated for true thickness values of less than 48 μm.

### Visualization of vascularization

**Figure 4:**
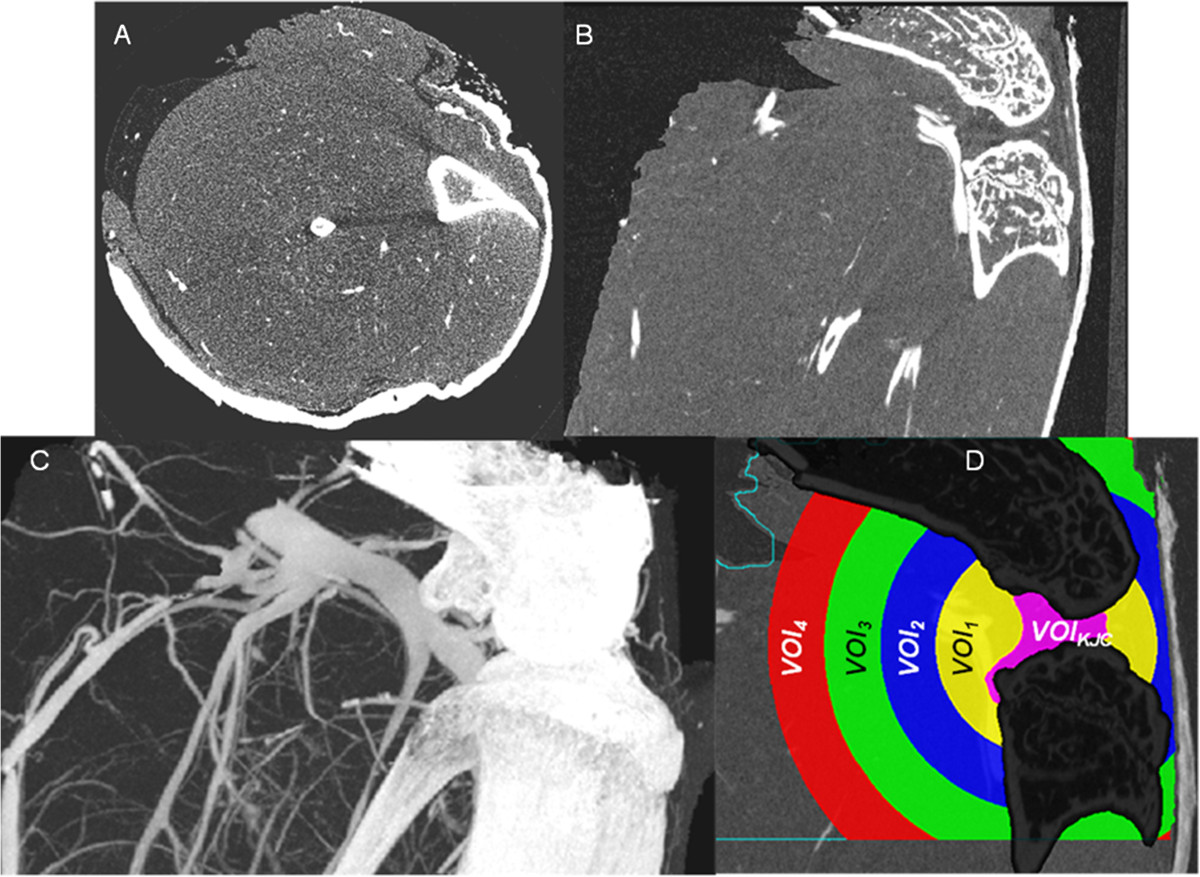
**μCT images of a knee joint of a non-arthritic mouse perfused with lead containing contrast agent. (A)** Axial and **(B)** oblique coronal multiplanar reformations (MPR). **(C)** Maximum intensity projection (MIP) showing similarity in image contrast of bone and contrast agent filled blood vessels. Beam hardening artifacts, which complicate vessel segmentation, are visible as dark bars between tibia and fibula in **(A)**. **(D)** Separation of volume of interest (VOI)_Capsule_ into four different analysis VOIs 1–4.

Axial and oblique coronal multi-planar reformations (MPR) of a μCT dataset of a knee joint are shown in Figure 4. Some of the blood vessels perfused with lead- containing contrast agent can be recognized but the vascular tree of a normal non-arthritic wild-type mouse becomes only visible in the maximum intensity projection (MIP). The technique to ‘remove’ the bones and to segment the vessel is described in the Appendix. Figure 4D shows the four VOIs (1–4) used for the vessel quantification, representing concentric sphere shells positioned around the center of the segmented knee joint cavity (VOI_KJC_). In each of the four analysis VOIs the parameters described in Table 1 were measured.

### Comparison of non-arthritic and arthritic mice

**Figure 5:**
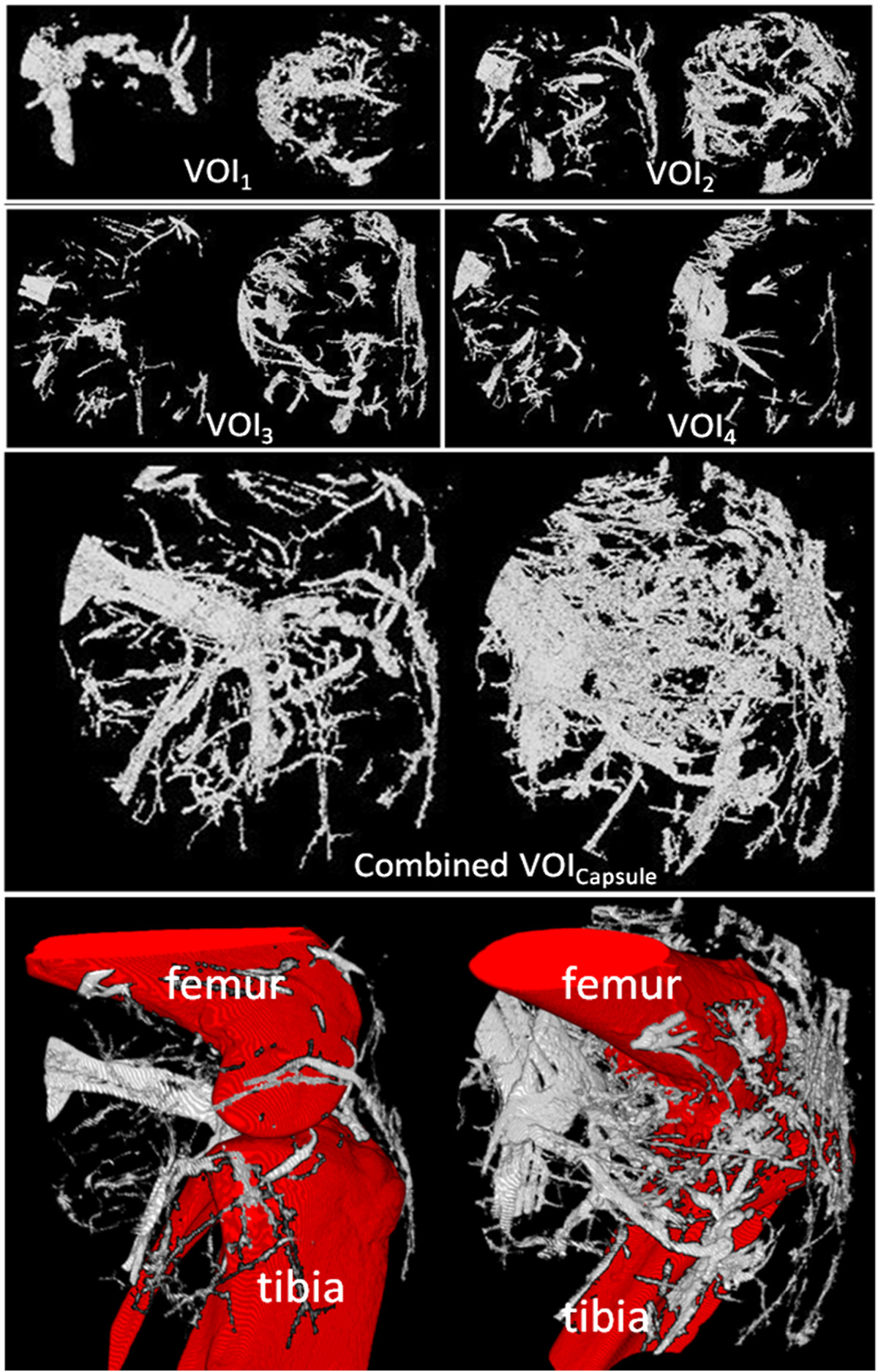
**Vessel segmentation results using MSLAT algorithm in the four different volumes of interests (VOIs) 1–4 and in the combined VOI**_**Capsule**_ **.** In each box the results for a non-arthritic wild-type control (left images) and an arthritic human tumor necrosis factor alpha transgenic (hTNFtg) mouse (right images) are shown. The combined VOI_Capsule_ is shown with and without femur, tibia and fibula. Also please note that the images of VOIs 1 – 4 are scaled differently.

**Figure 6:**
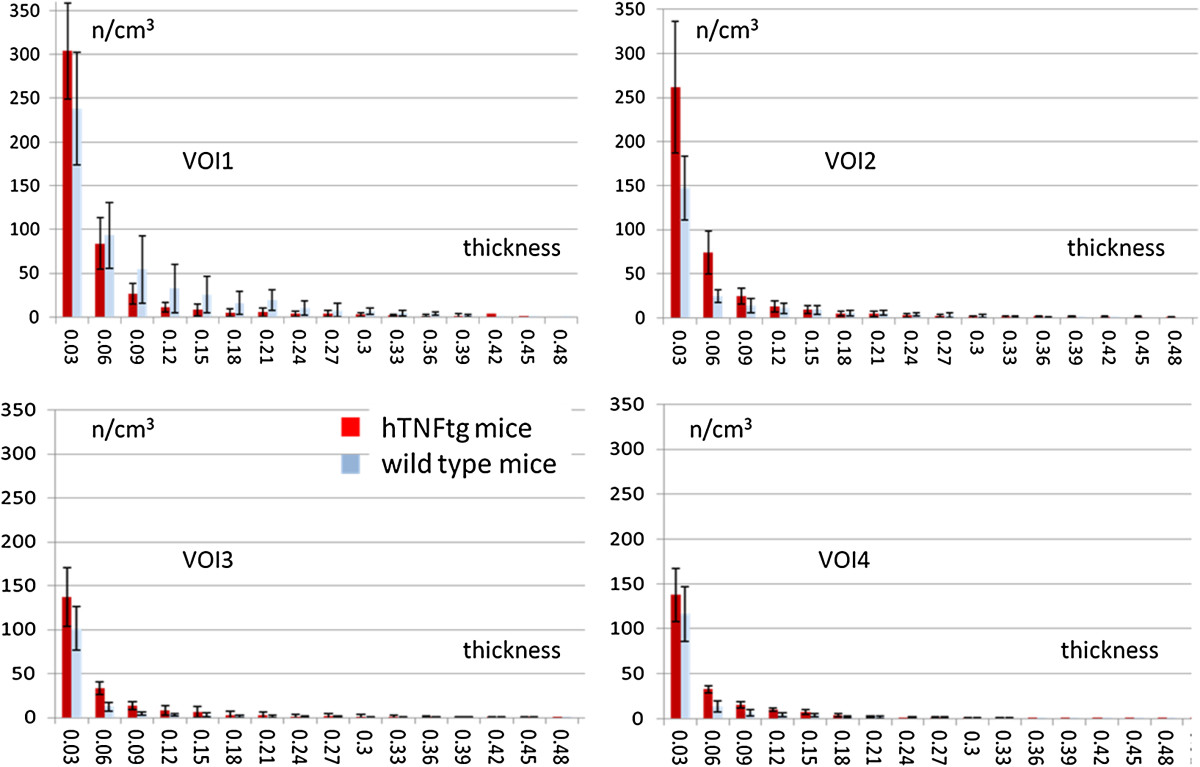
**Vessel thickness distribution (mean ± SD) in volume of interests (VOI ) 1–4, averaged over all samples in each group and normalized with the corresponding total volume (TV).** Red bars: arthritic human tumor necrosis factor alpha transgenic (hTNFtg) mice; blue bars: non-arthritic wild-type controls.

Finally, we compared the vascular tree of non-arthritic wild type mice (controls) and arthritic hTNFtg mice. Representative volume renderings for each of the four VOIs in non- arthritic wild type mice and arthritic hTNFtg mice are shown in Figure 5. Vessel density and number decreased in both strains from the most central VOI_1_ to the most peripheral VOI_4_, (Table 2). This effect is independent of vessel thickness; meaning that the closer the VOI is located to the joint, the higher the vascularization is (Figure 6). Furthermore, in all four VOIs the number of smaller vessels was consistently larger than the number of larger vessels but obviously larger vessels have a higher impact on *VV/TV* than smaller vessels.

**Table 2:** **Vascular parameters in non-arthritic and arthritic mice**

		*VOI*_*1*_	*VOI*_*2*_	*VOI*_*3*_	*VOI*_*4*_
*TV [mm* ^*3*^ *]*	WT	10.6 ± 5.2	34.6 ± 4.9	59.6 ± 4.6	69.0 ± 7.0
	hTNFtg	11.5 ± 3.1	40.0 ± 11.3	66.0 ± 13.4	78.4 ± 14.4
*VV/TV [%]*	WT	5.9 ± 3.1	2.0 ± 0.6^2^	1.1 ± 0.5^1^	1.4 ± 0.5^2^
	hTNFtg	3.7 ± 1.8	3.2 ± 0.7	1.8 ± 0.4	2.4 ± 0.3
*VS [mm* ^*2*^ *]*	WT	6.2 ± 3.7	8.4 ± 3.0^2^	9.4 ± 3.8^1^	12.5 ± 5.0^2^
	hTNFtg	6.0 ± 1.9	18.7 ± 7.9	16.0 ± 5.6	22.3 ± 5.4
*V.Th*_*3D*_ *[μm]*	WT	81.4 ± 19.5^1^	71 ± 14.6	56.2 ± 10.2	57.4 ± 7.1
	hTNFtg	59.0 ± 13.9	61 ± 9.1	65.0 ± 21.5	64.0 ± 6.5
*V.N*_*2D*_ *[1/mm]*	WT	1.4 ± 0.3	0.9 ± 0.2^3^	0.8 ± 0.1^2^	0.8 ± 0.1^2^
	hTNFtg	1.6 ± 0.2	1.5 ± 0.2	1.0 ± 0.1	1.0 ± 0.1
*V.Sp*_*2D*_ *[mm]*	WT	0.6 ± 0.2	0.9 ± 0.2^3^	1.1 ± 0.2^2^	1.1 ± 0.2^1^
	hTNFtg	0.5 ± 0.1	0.6 ± 0.1	0.9 ± 0.1	0.8 ± 0.1

TV: total volume; VV: vessel volume; VS vessel surface; V.Th_3D_: vessel thickness; V.N_2D_: vessel number; V.Sp_2D:_ vessel spacing; WT: wild type mice; hTNFtg: human tumor necrosis factors transgenic mice.

^1^0.01 ≤ p < 0.05. ^2^0.001 ≤ p <0.01; ^3^p < 0.001.

In VOI_2_, VOI_3_, and VOI_4_ average vessel density, vessel surface and vessel number were significantly increased in arthritic hTNFtg mice and vessel spacing was significantly decreased compared to the wild type controls (Figure 6). In VOI_2_, VOI_3_, and VOI_4_ vessel thickness did not differ significantly between the two strains (Table 2) although numerically thickness was higher in arthritic hTNFtg mice than in controls. In VOI_1_, which is closest to the knee joint results differed. Results for average *VV/TV*, *VS*, *V.N*_*2D*_, and *V.Sp*_*2D*_ were no longer significantly different between the two strains (Table 2). Average *VV/TV* was numerically 50% larger in the wildtype controls and average *V.Th*_*3D*_ in VOI_1_ was even significantly larger in the wildtype controls, suggesting profound reorganization of the vascular tree during arthritis. Further insights were taken from the analysis of vessel thickness in VOI_1._ The central joint compartment contained more small (30–45 μm) vessels in the hTNFtg mice than in controls but the number of thicker (>60 μm) vessels is higher in the controls, explaining the higher average *VV/TV* value for VOI_1_ in the controls.

### Precision errors and computing time

Reanalysis precision results are shown in Table 3. Precision errors varied between 1% and 6%. Typically inter-operator precision errors were higher than intra-operator errors, which rarely exceeded 3%. High inter-operator errors were found in VOI_1_ for VV/TV and VS, which indicates differences in manual corrections of vessel segmentation close to the bone surface, since in VOI_1_ the proportion of bone surface is higher than in the other three VOIs. Average analysis time for a 800^3^ voxel dataset (size 1.4 GB) including potential manual corrections was 15 min on a 3.4 GHz Intel Xeon 4 core PC with 16 GB of memory.

**Table 3:** **CV**_**RMS**_ **in % for inter- and intraoperator analysis**

		*VOI*_*1*_	*VOI*_*2*_	*VOI*_*3*_	*VOI*_*4*_
TV [mm^3^]	Inter	2.5	2.2	1.5	1.7
	Intra	1.6	0.6	1.0	1.6
VV/TV [%]	Inter	6.0	3.0	2.8	4.7
	Intra	3.3	2.0	2.6	2.5
VS [mm^2^]	Inter	6.0	2.9	4.1	4.9
	Intra	5.0	2.5	2.0	4.1
V.Th_3D_ [μm]	Inter	3.1	1.8	3.7	4.7
	Intra	2.4	1.8	1.3	1.3
V.N_2D_ [1/mm]	Inter	2.8	1.8	5.6	2.8
	Intra	2.2	1.8	1.8	2.2
V.Sp_2D_ [mm]	Inter	3.9	2.2	5.0	3.1
	Intra	2.7	2.0	2.0	2.6

TV: total volume; VV: vessel volume; VS vessel surface; V.Th_3D_: vessel thickness; V.N_2D_: vessel number; V.Sp_2D:_ vessel spacing.

## Discussion

We presented an integrated framework for high-resolution μCT imaging and analysis of vascularization in the normal and arthritic knee joint of the mouse. Compared to other studies the main advantage of this technique is the fully 3D imaging and analysis approach. The application of lead containing contrast agent in combination with a hybrid segmentation technique and the virtual removal of bone tissue allows for a reliable separation of the blood vessel system from bone and soft tissue.

Recently several studies have used comparative approaches applying μCT in vitro in mice perfused with Microfil to study angiogenesis in tumors. [10, 11] which also demonstrated the potential of this in vitro technique. One additional complication in our application was the comparable contrast of perfused vessels and bone which required the use of a hybrid segmentation approach. The presence of bone also prevents the effective use of advanced fluorescence based 3D analysis techniques used in tumor research [17].

We tested the integrated framework developed here for the analysis of vascularization in an animal model of inflammatory arthritis, which is characterized by increased vasculogenesis. Indeed, this approach was sensitive enough to detect the increased vascularization elicited by arthritis. Independent of vessel thickness, vessel density was increased in arthritic hTNFtg mice compared to non-arthritic controls. Consistent with this finding vessel surface and vessel number were increased, whereas vessel spacing was decreased in arthritis. Stunningly, the distribution of larger and smaller size vessels was fundamentally different between arthritic and non-arthritic mice.

Technically, we have overcome several hurdles in analyzing the microvascular tree of arthritic joints in mice. First, the combination of two different segmentation algorithms improved the overall segmentation result at acceptable computational performance. While still some user interaction was necessary to edit the vessel segmentation intra-operator reanalysis precision errors were low and interoperator reanalysis precision errors were acceptable. Nevertheless, operator interactions should be further decreased to improve reanalysis precision. Second, the size of the VOIs was adjusted to the size of the knee, which facilitates the analysis of cross-sectional studies involving mice of different sizes. Importantly, total VOI volume did not differ significantly between arthritic and non-arthritic mice for any of the four different VOIs.

Finally, another important part of the study was the use of digital vessel models to validate the quantitative parameters. None of the segmentation techniques was perfect. MS showed lower accuracy errors for vessel thickness and volume but higher accuracy errors for vessel number than LAT. Also, one has to consider that due to the blurring step applied to the digital models one cannot expect to exactly measure the true vessel thickness and volume used in the simulation. One cannot correct the limited spatial resolution of a CT scanner and hence resulting problems like partial volume effects are hampering “true” segmentations. Therefore, highest accuracy errors were measured for vessels that were smaller than twice the minimum voxel.

**Figure 7:**
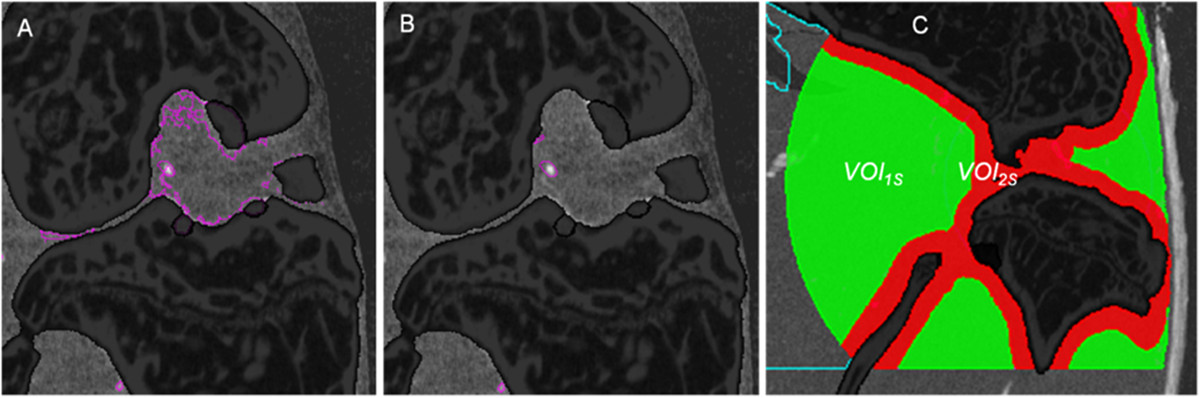
**Incorrect vessel classification. (A)** Soft tissue classified as vessel close to the bone surface due to beam hardening effects using local adaptive threshold (LAT) segmentations. **(B)** The multi-scale (MS) approach largely avoids the wrong classification. **(C)** In the final hybrid algorithm developed for this study the LAT approach was used in VOI1S (green) and the MS approach in VOI2S (red).

One limitation of the study was that the combination of the LAT and MS segmentation and in particular the definition of the VOIs used for one of the two algorithms (Figure 7) as well as the separation of the joint capsule into 4 concentric VOIs was empirical. Also the vessel segmentation close to the bone was still not perfect and required user corrections in about 50% of the cases. No attempts have been integrated into our framework to improve the connectivity of the vessel network, for example by joining branch ends that are close together. Thus, measures of connectedness of the vessel tree other then the number of vessels were not obtained. Our technique could be used to quantify vessel branching but this has not been implemented yet. Another technique recently developed for the vessel quantification in tumors is multispectral fluorescence ultramicroscopy using optical sectioning [17], however, this technique is difficult to use in the presence of bone shading vessels behind bone. Alternative techniques such as μMRI do not have the required resolution. More indirect, MR methods like DCE or perfusion have not been published for the mouse knee principally due to the above mentioned limitation of spatial resolution.

Finally we did not compare the μCT results with conventional histological procedures which often are still considered as gold standard when morphologically characterizing vessel density and vessel size. However, as vessel morphology may vary from slice to slice a real comparison would require a 2D-3D registration or a comparison with 3D histology, which was beyond the scope of the current study. Instead we used digital models for the validation process.

## Conclusions

A fully 3D imaging and analysis framework has been developed to quantify the vascularization in the mouse knee. The complete knee, i.e. the total joint capsule was included in the investigation. The segmentation using a hybrid approach of LAT and MS techniques was largely automated and allowed for operator corrections, which may be necessary in the vicinity of the bone tissue. Standard morphological parameters such as *VV/TV*, *VS*, *V.Th*_*3D*_ and *V.Sp*_*2D*_ were used to assess the vascular network within anatomically defined VOIs centered in the knee joint and covering the complete knee joints capsule. Intra- and interoperator analysis precision was good (<3%) and acceptable (<6%) and accuracy was validated with simulated digital vessel models, suggesting that this framework is useful to analyze the changes of the microvascular architecture in arthritis.

## Appendix: vessel segmentation

### Preparative steps

The novel vessel segmentation technique introduced in this study is a multi-step procedure consisting of bone segmentation, definition of a volume of interest (VOI) and vessel segmentation inside this VOI. Due to the similarity of the intensity values of bone and contrast agent filled vessels and due to noise and partial volume and beam hardening artifacts in the vicinity of bone (Figure 4), the first step is the virtual removal of the bones from the investigation volume. An alternate approach often used in specimen studies is a decalcification of the bone matrix. But this is a tedious time consuming technique. Dedicated software tools such as the one developed in this study offer the possibility to virtually remove the bone before the vessel segmentation. After a coarse segmentation of the larger vessels, bone was segmented using an adaptive intensity threshold based volume growing algorithm and subsequent morphologic operations as described earlier [18]. The initial coarse vessel segmentation step is required to prevent the volume growing process used for the bone segmentation from leaking out into the larger vessels.

The next step is the definition of a VOI to constrain the vessel analysis. The size and position of the VOI should be defined relative to the anatomy of the individual mouse knee in order to be able to compare results in cross-sectional studies. For longitudinal in vivo studies, which are not topic of this investigation, also a good reproducibility of the VOI position is required. During RA propagation the inflammatory process is concentrated within the synovium. Therefore, it is useful to position the VOI in the centre of the knee and define its size as a portion of the size of the bounding sphere covering the knee joint cavity.

**Figure 8:**
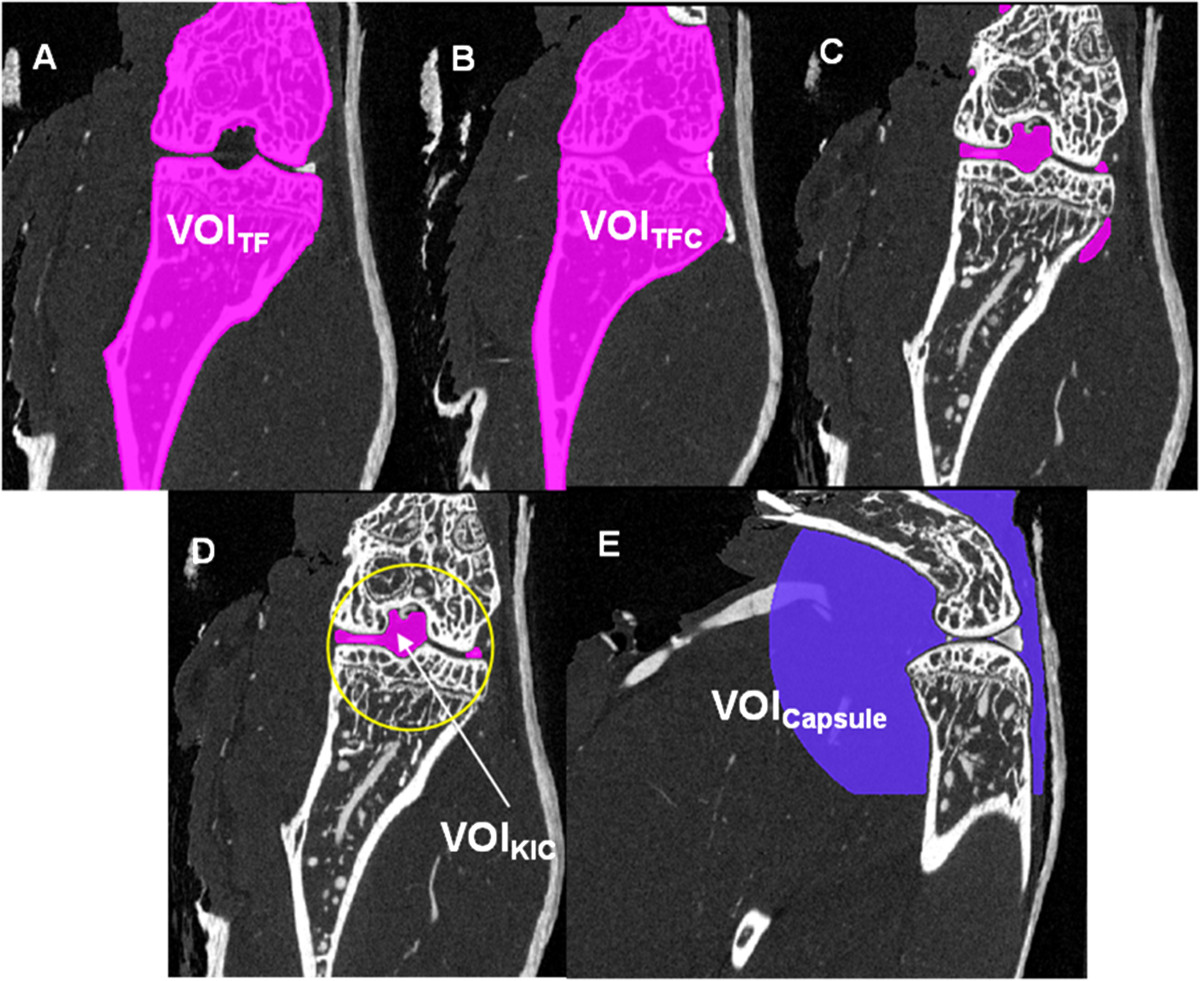
**Definition of volumes of interest (VOI) and segmentation of the knee joint cavity. (A)** Combined tibia and femur VOIs (VOI_TF_), **(B)** after closing VOI_TF_ with a big structure element (VOI_TFC_), **(C)** resulting preliminary knee joint cavity after subtraction of VOI_TF_ from VOI_TFC_, **(D)** largest connected volume defined as final knee joint cavity (VOI_KJC_) with bounding sphere, **(E)** resulting analysis VOI_Capsule_.

For the required segmentation of the knee joint cavity, the segmented tibia and femur VOIs were combined into one binary volume (VOI_TF_) and closed with a big structure element resulting in the binary volume VOI_TFC_ (Figure 8). The binary volume of the knee joint cavity (KJC) can then easily be obtained by the subtraction:

$$\mathrm{VO}I_{\mathrm{KJC}}=\mathrm{VO}I_{\mathrm{TFC}}\backslash \mathrm{VO}I_{\mathrm{TF}}$$

in combination with a subsequent search and selection of the largest connected volume. The bounding sphere was a sphere positioned in the VOI_KJC_ centre and fully included VOI_KJC_. It was scaled with an empirically defined factor of 2.5 to approximate the joint capsule, which is not visible in the μCT datasets. The resulting VOI_Capsule_ was used as VOI for the vessel segmentation (Figure 8). During RA the volume of the joint capsule increases, resulting in a swollen joint. However, in our analysis VOI_Capsule_ does not depend on disease status.

### Volume growing approach using local adaptive threshold (LAT)

After virtual removal of the bones, in VOI_Capsule_ vessels must be separated from soft tissue. Unfortunately due to partial volume artifacts thicker vessels have higher intensity values than thinner ones, which are difficult to segment in the presence of noise. As a consequence, the use of global thresholds for segmentation results in discontinuities in the vessel network. As an alternative we used a local adaptive segmentation, which easily adapts to the changing intensity values in a given neighbourhood. First, two thresholds were computed:

$$T_{\mathrm{low}}=\mu_{\mathrm{ST}}+2\cdot \sigma_{\mathrm{ST}}$$

$$T_{\mathrm{high}}=0.65\cdot H_{\max}$$

where μ_ST_ is the mean soft tissue value, σ_ST_ the standard deviation and H_*max*_ the maximum value of a Gaussian curve fitted to the histogram of the intensity values of the voxels in VOI_Capsule_. If the intensity value lies outside the interval [T_*low*_, T_*high*_] the voxel can be classified as a soft tissue or vessel voxel.

$$f\left(I_{\mathrm{xyz}}\right)=\left\{\begin{array}{cc} \mathrm{if}\;I_{\mathrm{xyz}}<T_{\mathrm{low}} & \mathrm{soft}\;\mathrm{tissue}\\ \mathrm{if}\;I_{\mathrm{xyz}}\geq T_{\mathrm{high}} & \mathrm{vessel}\\ \mathrm{otherwise} & g\left(\mu ,\;\sigma \right) \end{array}\right.$$

In the ‘otherwise’ case the following adaptive classification is used:

$$g\left(\mu ,\sigma \right)=\left\{\begin{array}{cc} \mathrm{if}\;\sigma /\mu <\alpha \cdot \tau & \mathrm{vessel}\\ \mathrm{otherwise} & \mathrm{soft}\;\mathrm{tissue} \end{array}\right.$$

$$\tau =\sigma_{\mathrm{ST}}/\mu_{\mathrm{ST}}$$

α=1.0

where *μ* and *σ* are the mean intensity and standard deviation of the 26-neighbourhood of the voxel under consideration. All connected voxels satisfying the criterion above will be classified as vessel voxels using again a volume growing algorithm (Figure 3A). The method described here does not use any vessel form or shape properties. It only searches for homogeneously connected voxels of high intensity. In order to use a priori information of vessel properties, in addition a multi-scale segmentation method was implemented and compared to volume growing with local adaptive thresholds.

### Multi-scale segmentation (MS)

Based on earlier studies on vessel segmentation [19, 20] a multi-scale algorithm was used to enhance the vessel contrast prior to segmentation. It is based on the assumption that vessels are of cylindrical shape and that highest voxel intensity values occur in the vessel center. To minimize noise and increase vessel intensity, an iterative Gaussian smoothing with different mask sizes was used. After each iteration step a response function *R*_*s*_ was determined [20] from the three eigenvalues *λ*_*1*_*- λ*_*3*_ of the Hessian matrix and the maximum value of each iteration was saved as result.

$$R_{S}\left(\lambda_{1},\lambda_{c}\right)=\left\{\begin{array}{cc} e^{\left(-\frac{\lambda_{1}^{2}}{2{\left(\alpha_{1}\lambda_{c}\right)}^{2}}\right)} & \lambda_{1}\leq 0,\;\lambda_{c}\neq 0\\ e^{\left(-\frac{\lambda_{1}^{2}}{2{\left(\alpha_{2}\lambda_{c}\right)}^{2}}\right)} & \lambda_{1}>0,\;\lambda_{c}\neq 0\\ 0 & \lambda_{c}=0 \end{array}\right.$$

$$\lambda_{c}=\min\left(-\lambda_{2},-\lambda_{3}\right)$$

$$\alpha_{1}=0.5;\;\alpha_{2}=2.0$$

$$\lambda_{1}\geq \lambda_{2}\geq \lambda_{3\;\text{.}}$$

In this study the ITK implementation of *R*_*s*_ was employed. After contrast enhancement the vessels were determined using a K-mean classifier (Figure 3B) [21].

### Hybrid MSLAT algorithm

**Figure 9:**
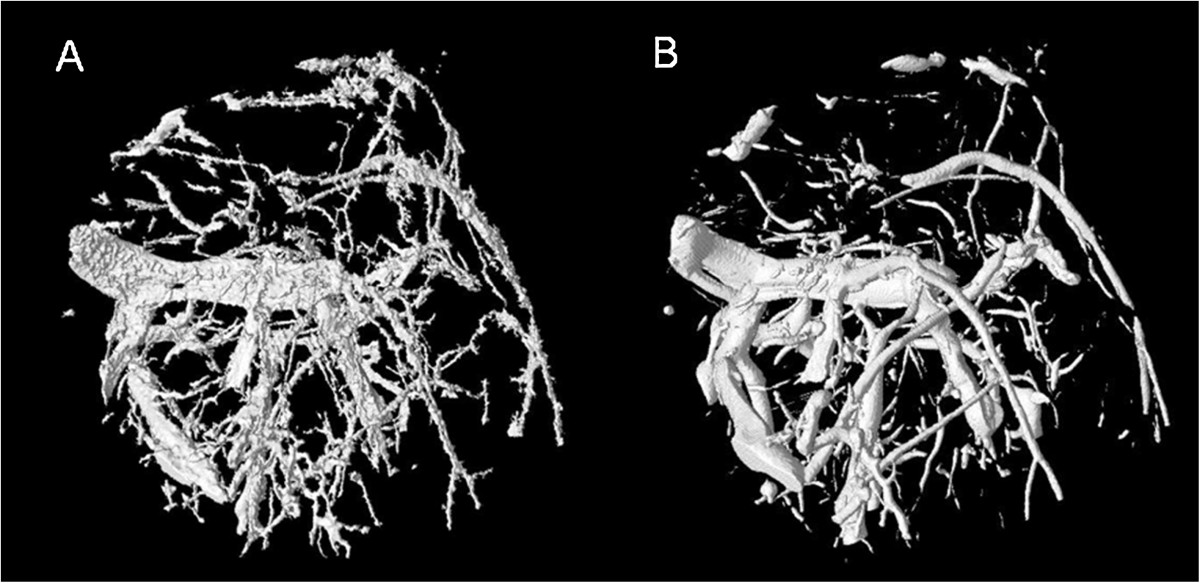
**Result of vessel segmentation. (A)** Volume growing based on local adaptive thresholds; **(B)** Multi-scale based segmentation method.

The LAT approach can easily be implemented and computed. Results are adapted to changing intensity values and LAT shows better vessel connectedness than the MS algorithm (Figure 9). However, due to beam hardening artifacts the intensity values of soft tissue voxels in the vicinity of bone are frequently comparable to those of bone (Figure 7A). Here LAT very often fails and the MS algorithm shows better results (Figure 7B). Therefore, we used a combination of the two methods to improve the overall result. Consequently VOI_Capsule_ was divided into two VOIs (Figure 7C). LAT segmentation was applied in VOI_1S_ (green) and MS segmentation in VOI_2S_ (red).

## Authors’ original submitted files for images

Below are the links to the authors’ original submitted files for images. Authors’ original file for figure 1 Authors’ original file for figure 2 Authors’ original file for figure 3 Authors’ original file for figure 4 Authors’ original file for figure 5 Authors’ original file for figure 6 Authors’ original file for figure 7 Authors’ original file for figure 8 Authors’ original file for figure 9

## Abbreviations

BV/TV: Bone volume per tissue volume
CT: Computed tomography
CV_rms_: Root mean square coefficient of variation
hTNFtg: Human tumor necrosis factor transgenic
GT: Global threshold
KJC: Knee joint cavity
LAT: Local adaptive threshold
MIP: Maximum intensity projection
MPR: Multi-planar reformations
MS: Multi scale
MSLAT: Hybrid of MS and LAT segmentation
TV: Tissue volume
μCT: Micro-computed tomography
VOI: Volume of interest
V.N_2D_: Vessel number
V.S: Vessel surface
V.Sp_2D_: Vessel spacing
V.Th_2D_: Vessel thickness
V.Th_3D_: Vessel thickness calculated with direct method
VV: Vessel volume
VV/TV: Vessel volume per tissue volume.
