# Supplementary figures and images for: Characterization and quantification of angiogenesis in rheumatoid arthritis in a mouse model using μCT
Source: BMC Musculoskelet Disord. 2014 Sep 6;15:298. doi: 10.1186/1471-2474-15-298 (PMC4246538; doi:10.1186/1471-2474-15-298)

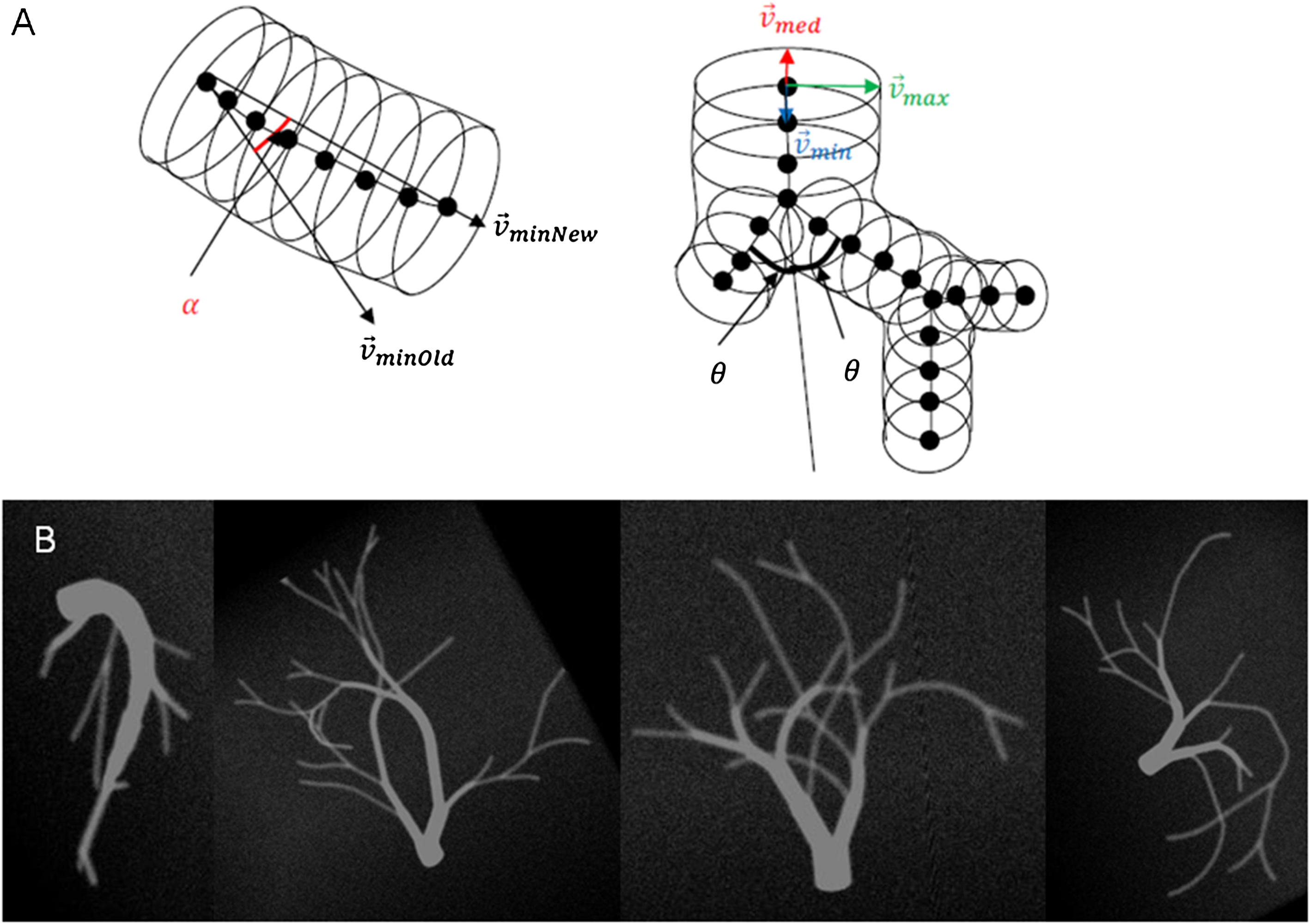

Supplement: Supplementary file 1 — Authors’ original file for figure 1 [file 12891_2014_2317_MOESM1_ESM.tif]

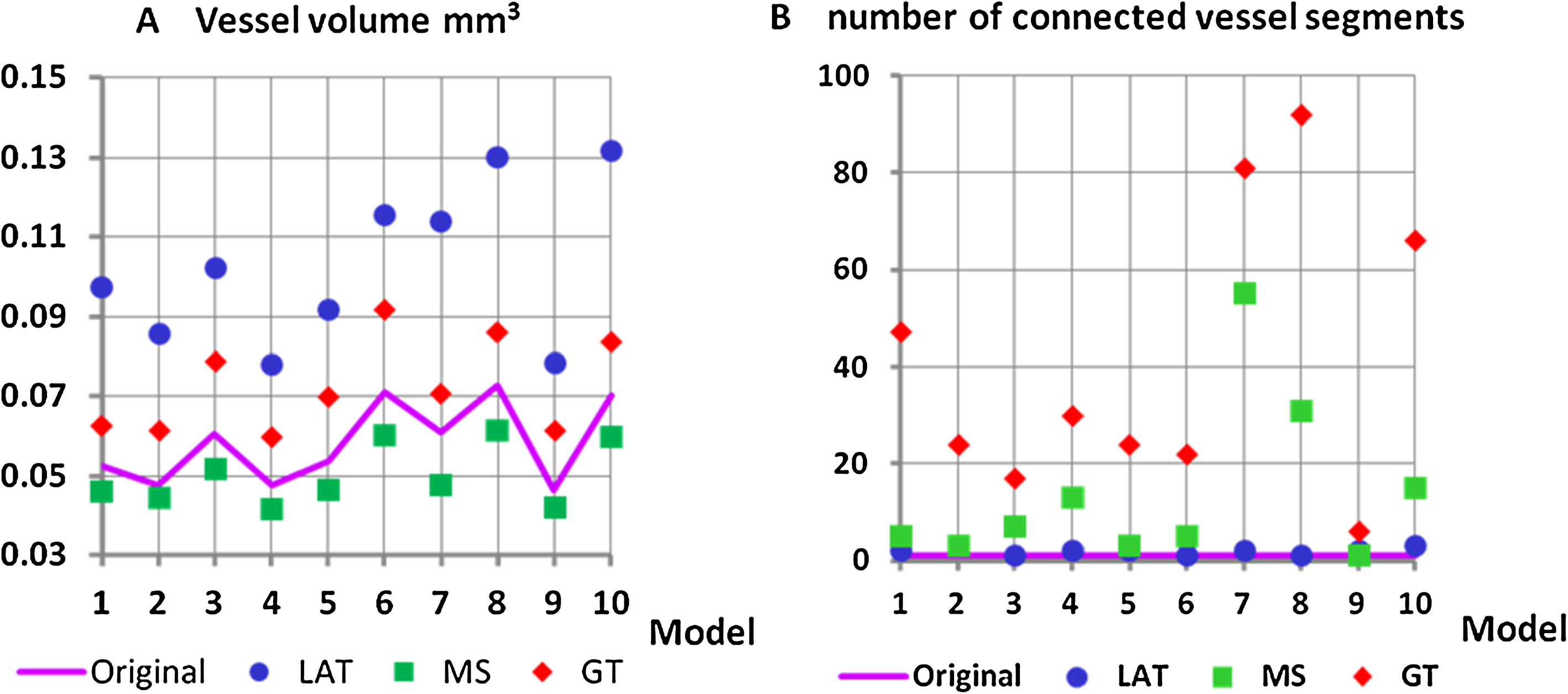

Supplement: Supplementary file 2 — Authors’ original file for figure 2 [file 12891_2014_2317_MOESM2_ESM.tif]

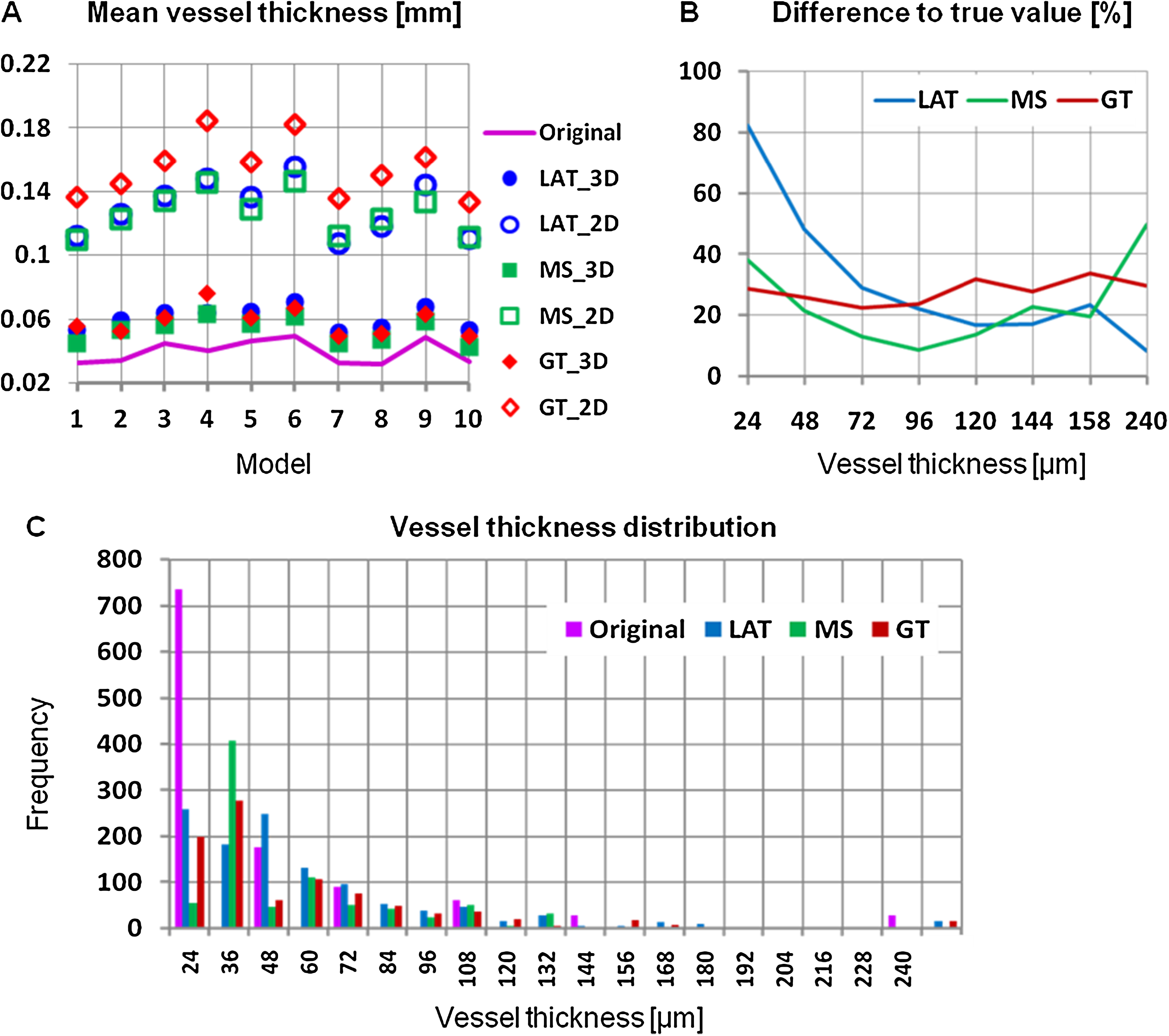

Supplement: Supplementary file 3 — Authors’ original file for figure 3 [file 12891_2014_2317_MOESM3_ESM.tif]

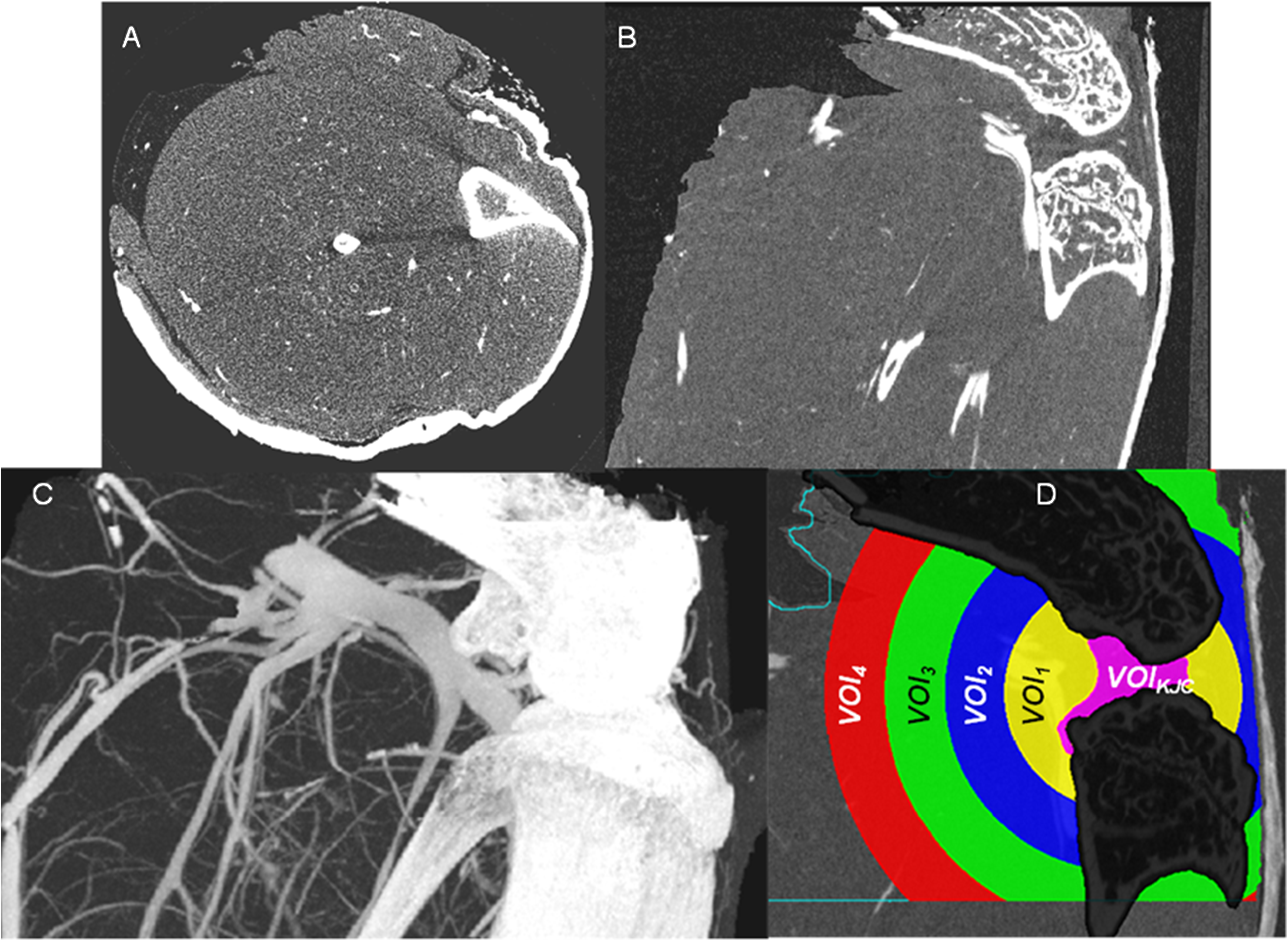

Supplement: Supplementary file 4 — Authors’ original file for figure 4 [file 12891_2014_2317_MOESM4_ESM.tif]

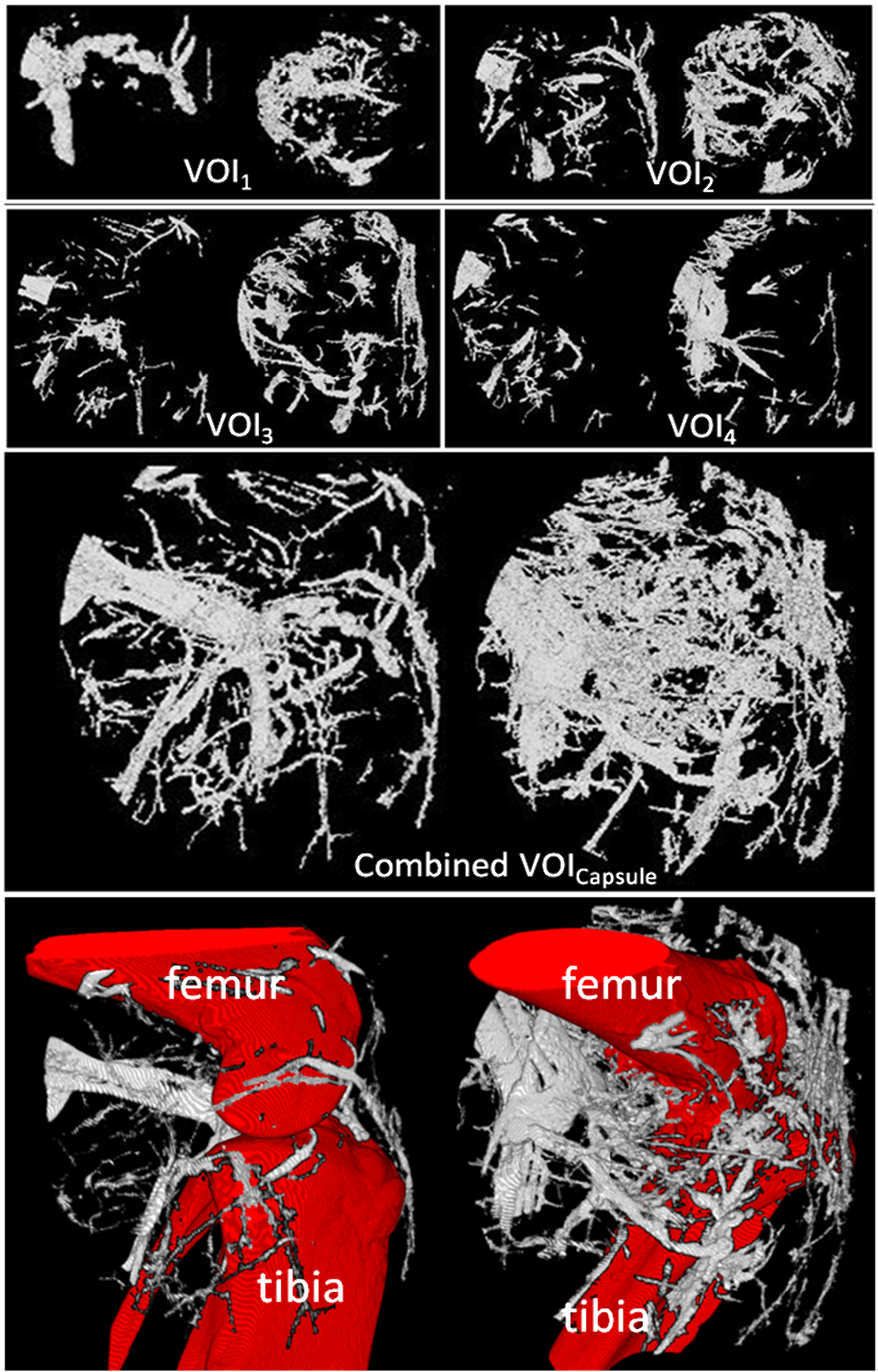

Supplement: Supplementary file 5 — Authors’ original file for figure 5 [file 12891_2014_2317_MOESM5_ESM.tif]

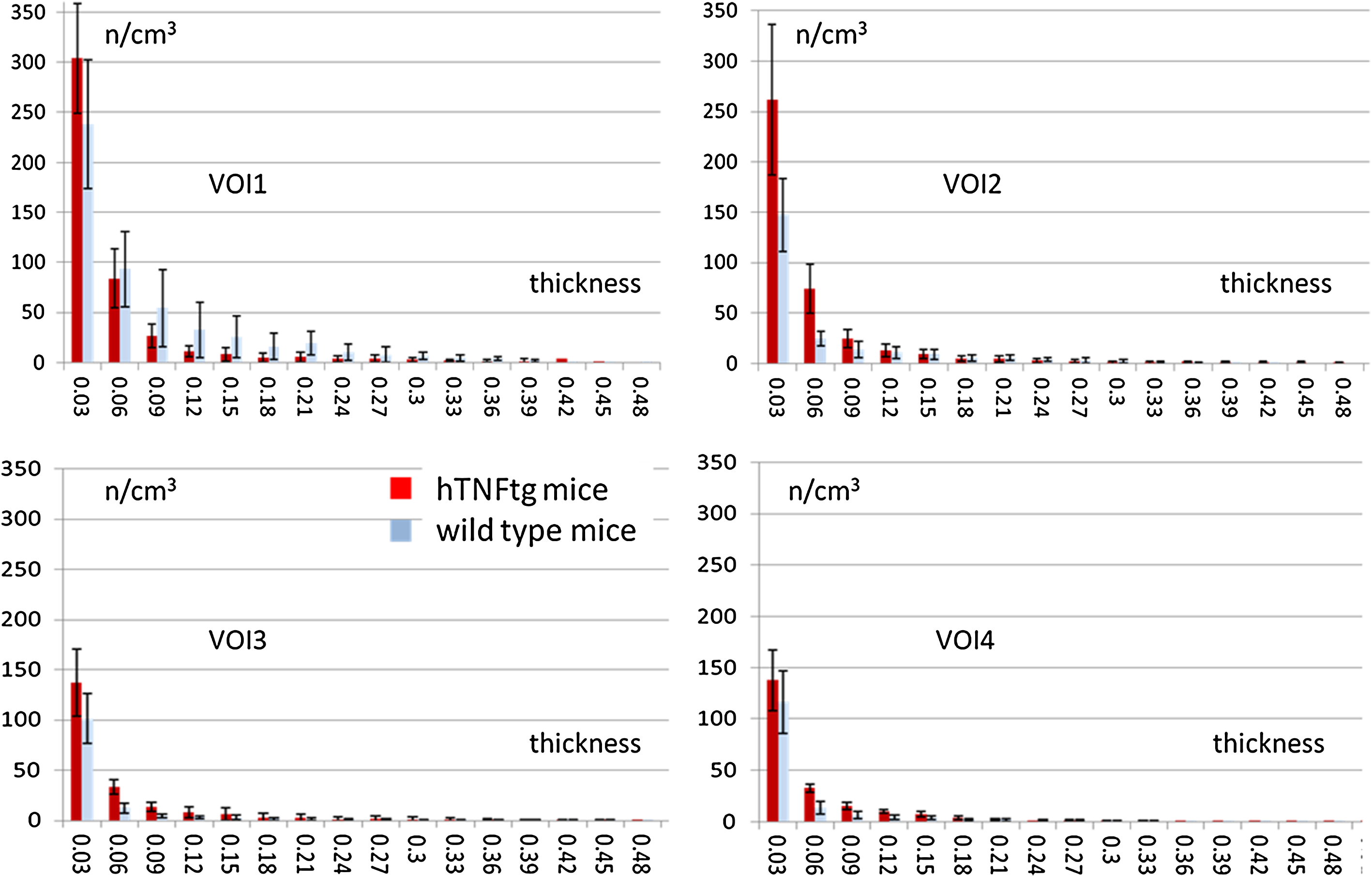

Supplement: Supplementary file 6 — Authors’ original file for figure 6 [file 12891_2014_2317_MOESM6_ESM.tif]

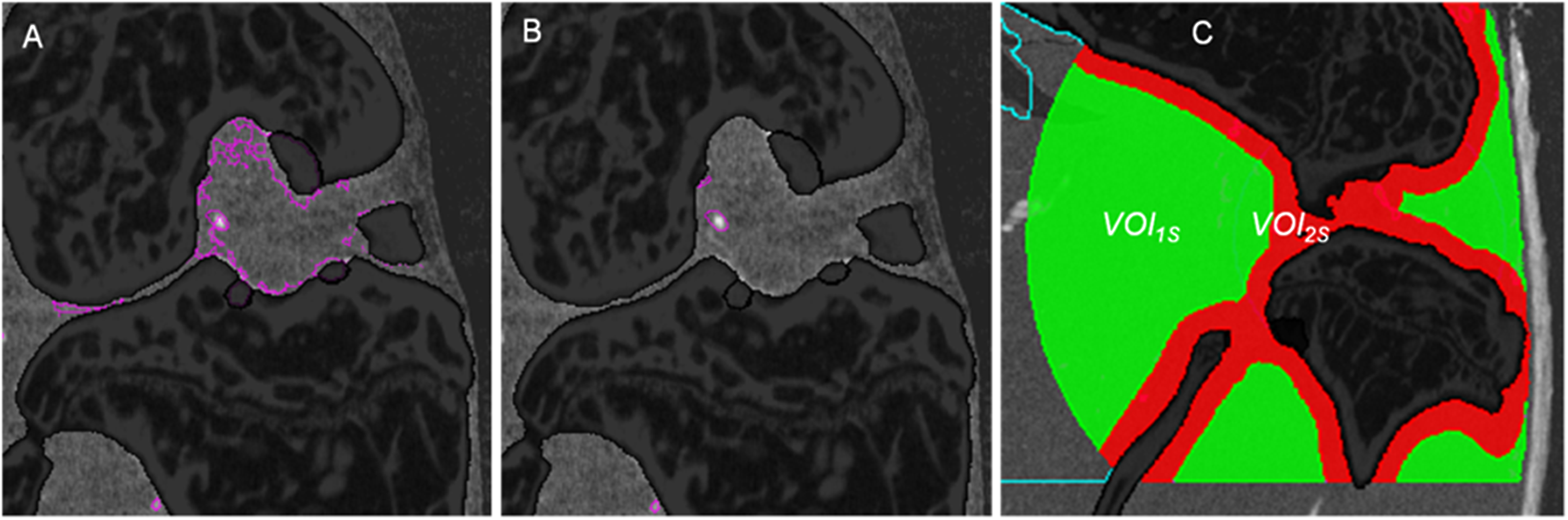

Supplement: Supplementary file 7 — Authors’ original file for figure 7 [file 12891_2014_2317_MOESM7_ESM.tiff]

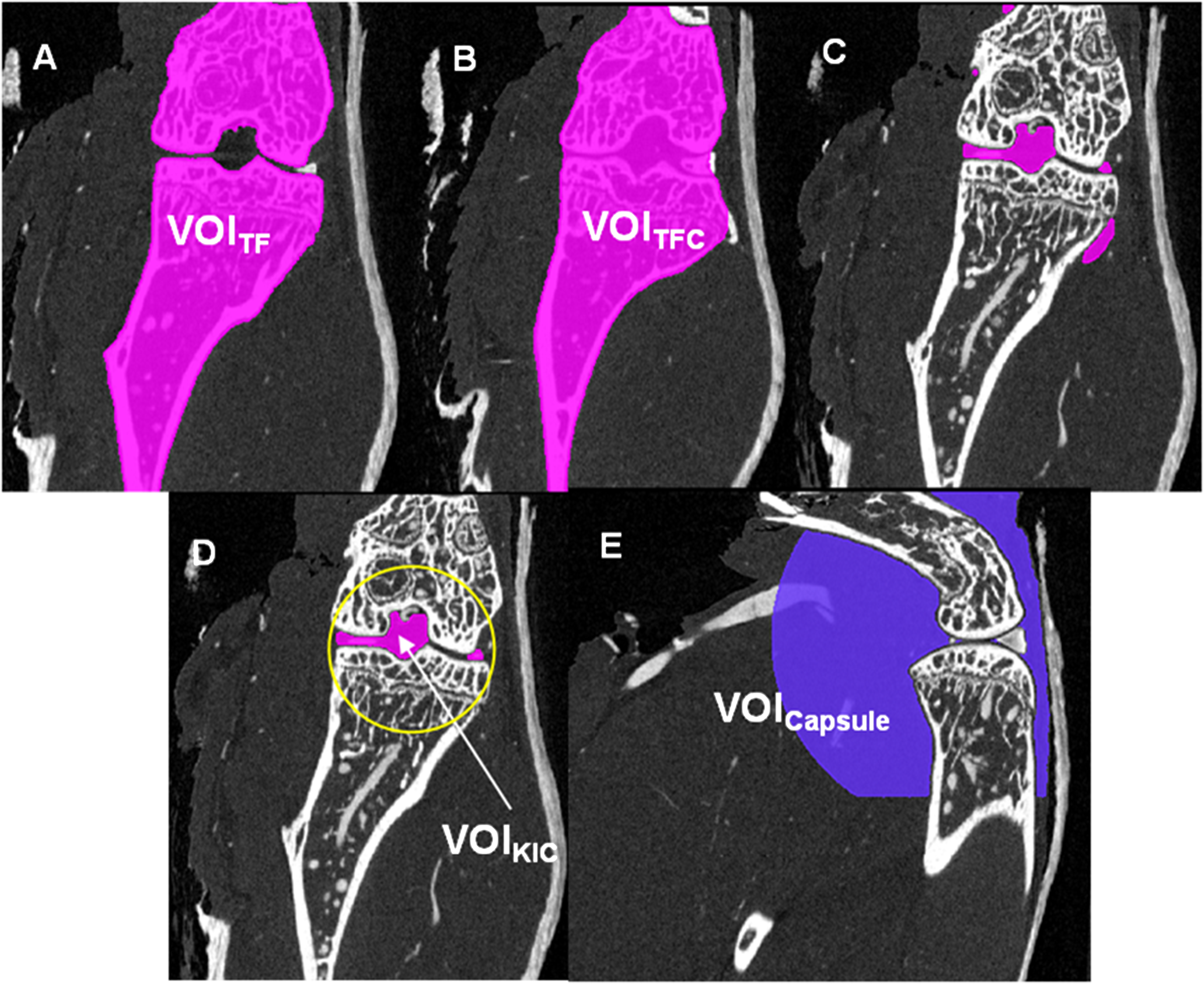

Supplement: Supplementary file 8 — Authors’ original file for figure 8 [file 12891_2014_2317_MOESM8_ESM.tiff]

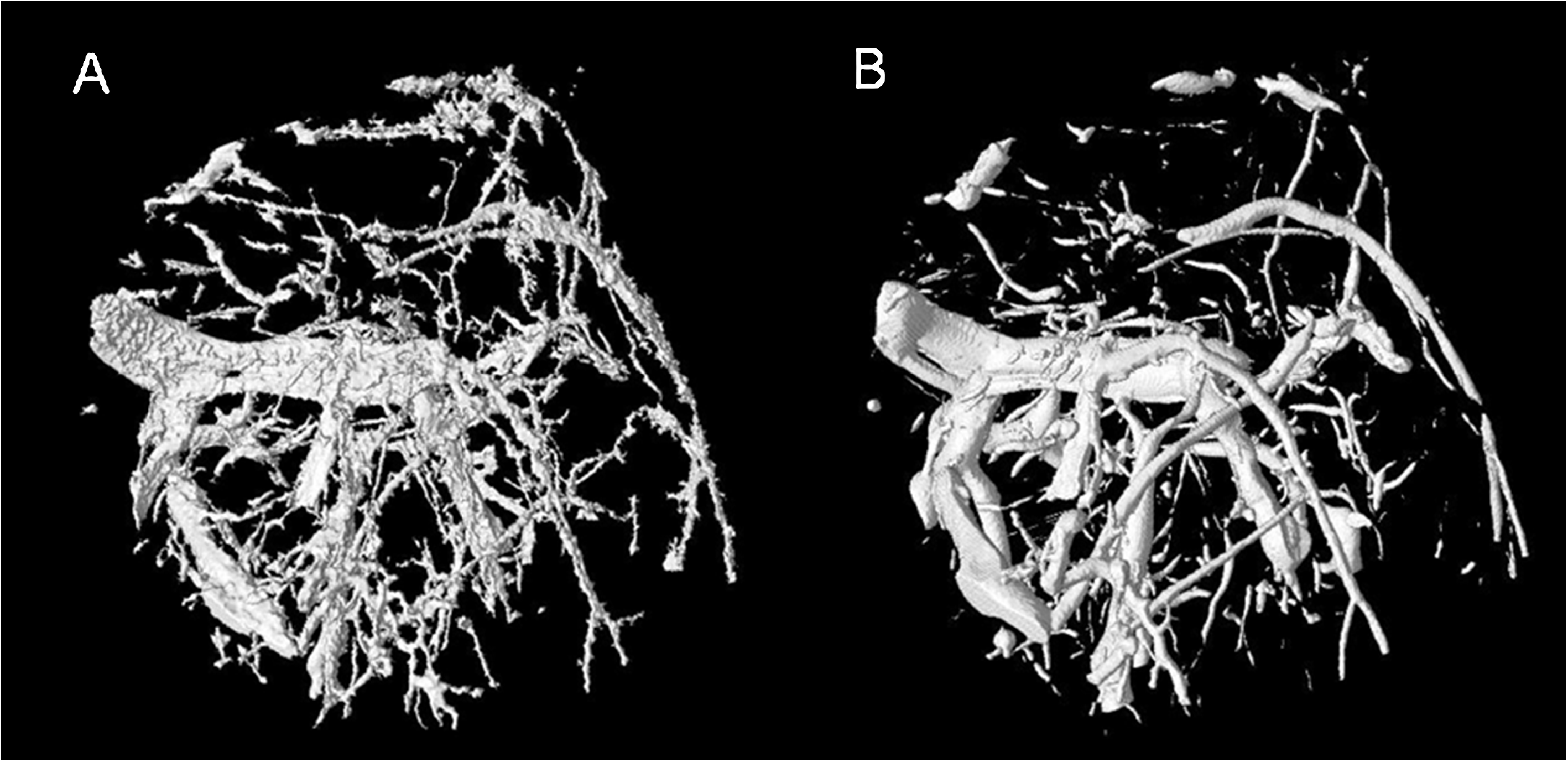

Supplement: Supplementary file 9 — Authors’ original file for figure 9 [file 12891_2014_2317_MOESM9_ESM.tiff]
